# A new method to compute mechanical properties of a standing skyline for cable yarding

**DOI:** 10.1371/journal.pone.0256374

**Published:** 2021-08-19

**Authors:** Christian Knobloch, Leo Gallus Bont

**Affiliations:** 1 Faculty of Environmental Sciences, Institute of Forest Utilization and Forest Engineering, Technische Universität Dresden, Tharandt, Germany; 2 Research Unit Forest Resources and Management, Research Group Sustainable Forestry, Swiss Federal Institute for Forest, Snow and Landscape Research WSL, Birmensdorf, Switzerland; China University of Mining and Technology, CHINA

## Abstract

Cable-based technologies are the backbone for logistics of timber or construction material on impassable terrain. In Central Europe, the use of standing skylines with pre-stressed, both-sided fixed-anchor cables and multi-span configurations with internal intermediate supports is common. To ensure a safe and cost-effective set-up for cable road operations, it is essential to identify and compute the properties of the skyline (e.g. load path, tensile forces). This task is challenging because it requires dealing with the non‐linear behaviour of the cable structure under the load and has to include all significant physical effects. Several approaches have previously been proposed as practical solutions, however not all physical effects were covered by those approaches, such as the inclination-dependent elastic prolongation of the cable or the longitudinal deflection of the sagging carriage. With our new proposed approach, we aim to close this gap of knowledge, and consider all relevant physical effects. We present a non-linear approach that is able to compute the properties of a wide range of standing skyline configurations, including those with additional cables. This approach offers an extensive solution and a flexible framework for considering individual configurations or particularities by adding equations to the equation system.

## 1. Introduction

Cable-based technologies are the backbone for timber harvesting as for logistics of construction material on steep slopes as on impassable lowlands. Not only in Central Europe, silvicultural and ergonomic restrictions heavily influence yarder configuration and design [1]. As a result, truck-mounted yarders with integrated processor heads and towers with a skyline fixed to anchors at both the head and tail spars (a standing skyline configuration) are most common in Central Europe. To ensure there is enough clearance from the ground, intermediate supports are frequently used, creating a multi-span configuration.

Both-sided, fixed-anchored skylines with multi-span configurations react sensitively to changes in cable tension. As a result, the operation of cable yarders is a dangerous job, with critical work accidents or deadly injuries occurring every year [2–7]. The general planning process of a single- or multi-span cable road is essential for a safe, cost-effective and environmentally sound operation.

To design a single- or multi-span cable line, one must fully understand the behaviour of a pretensioned as a loaded skyline. Therefore, accurate computation of the behaviour of the skyline, and furthermore of the combination of skyline and additional cables (mainline, haulbackline) are needed. Spinelli et al. [8] concluded that high-dynamic loads in a well-managed standing skyline operation are less frequent and extreme than expected. Therefore, it is also absolutely important for safety reasons to have a method that also accurately predicts the sag in order to enable a good and safe cable road/line design. That depends on the weight of the forwarded logs, the cable yarder power capacity, the ground profile, the anchors, the guylines, the location and height of the spars and the mounting tension force or minimal clearance required. The analysis and computation of the properties of the skyline and additional cables will result in the load path (in reference to the ground profile), the position of maximum deflection, the tensile forces of the unloaded and loaded skyline, the maximum load of the carriage, and the impact of changes in the surrounding temperature. It is a challenging task, as the non-linear behaviour of a loaded cable structure must be considered. Furthermore, optimization of the yarding process (e.g. using the minimum number of intermediate supports, an easy and quick set-up, and a safe and appropriately sized support system) will only succeed with detailed and exact knowledge of the parameters of the skyline [9]. Mologni et al. [10] criticize that most solutions for predicting tensile forces are based on a static approach, while in the field skyline tensile forces show a dynamic behaviour. As the dynamic oscillations of a cable are vibrating around the static state, the estimation of the static situation will be the basis for further examinations.

Several approaches to calculating skyline properties have been proposed as practical solutions. However, they are based on simplifications or approximations that avoid using the catenary, and they are mainly used to solve standard problems or show limitations in worst case situations. The aim of most of these approaches is to compute the sag at midspan (e.g. [11,12] or [13]) as an estimate of the maximum sag, but this is only true for spans on flat terrain. We hypothesize (and as [14] showed in his method) that with inclined spans, maximum sag is beside midspan position and with that the load path differs in a significant way (see Fig 5, point 2) and 3)).

To be used for Central European standing skyline configurations, these approaches must be able to handle both-sided fixed multi-span configurations and account for cable elasticity, the influence of mounting tension, and the location of intermediate supports. Only two of the established approaches fulfil these basic requirements: those introduced by [11] and [15] (Table 1). These approaches from the 1960s solve the non-linear behaviour of the catenaries with Taylor series approximations, and the solutions are useful only for end or midspan situations. The unstrained length of a skyline is constant, so elasticity or temperature change is the only reason why the sag of an unloaded both-sided fixed cable is changing. Beyond the approaches mentioned above, only those proposed by [14] and [16] (see Table 1) consider the influence of elasticity, although they do not consider multi-span configurations or the influence of mounting tension.

**Table 1:** Tabular summary of analysed approaches.

source	Calculation with help of the parabola (cable´s self-weight along secant)	Calculation with help of the catenary (system of non-linear equations, cable´s self-weight along cable)	Calculation of both- sided fix spans	Multi-span & both-sided fix spans	Influence of elasticity/tempera-ture	Multiple load	Calculation of any locations/tensions possible (not only worst case)	Influence of mainline, haulback-line	Friction at supports/movable cables	inclination dependent elastic prolonga-tion	Longitu-dinal deflection of the sagging carriage	Influence of mounting tension and their location
Stephan (1904) [21]	+											
Findeis (1923) [22]	+							(+)				
Hauska (1933) [20]	+						+	(+)	+			
Stüssi (1937) [25]		+	+				+					
Barat & Plawinski (1956) [11]	+	(+)	+				+					+
Zweifel (1960) [12]		(+)	+	+	+	+		(+)	+			+
Czitary (1961) [15]		(+)	+	+	+	+			+			+
Pestal (1961) [13]	+					+			+			
Carson & Mann (1970) [26]	+		(+)				+	+				
Carson & Mann (1971) [27]		+	+				+	+				
Carson (1977) [28]		+	+				+	+				
Irvine (1980) [32]		+	+		+						+	
Falk (1981) [29]		(+)										
Irvine (1981) [14]		+	+		+	+					+	
Kendrick & Sessions (1991) [30]	+		+									
Feyrer (1994) [24]	+	(+)					+					
Brown & Sessions (1996) [16]	+		+		+			+				
Dupire et al. (2015) [33]		+	(+)	+	+		+	+	+			(+)
Knobloch & Bont	+	+	+	+	+	+	+	+	+	+	+	+

The basic conditions for Central European cable yarder configurations are marked in grey.

Any simplifications used to solve the non-linear behaviour of the catenary in an easier way result in geometric and dimensional aberrations from the real situation that could lead to misinterpretation or, in the worst case, dangerous settings in the field, as described in [17].

Mologni et al. [10] obtained standing skyline properties with the help of finite element method simulations avoiding the usage of any formulas. The limitations of the finite element method are that the results of the simulation are strictly dependent of its boundary conditions (input data, number of elements, method of simulation), that detailed simulations requires experience how to set up the complex model design and that these simulations need long calculation time (as showed in [10]). At least, complex simulations requires an expensive software license.

The both-sided fixed sagging cable, a catenary, with a single or multiple load is an element of sophisticated engineering mechanics and is relevant in construction, the logistic and the forestry sectors.

The aim of this paper is to introduce a procedure to calculate the properties of standing skyline cable yarding systems with a system of non-linear equations. We propose that this approach will make it possible to:

1. easily adapt to variations in the setting by using a modular system;
2. calculate the catenary or the parabola for any location in any number of carriages in any multi-span configuration (adaptable for modelling special cases);
3. ignore or consider essential physical effects or influences such as temperature, slide over intermediate supports, longitudinal deflection, friction, additional cables, elastic guy lines, and inclination-dependent cable elasticity;
4. find an equilibrium to solve all unknown variables in a twostep procedure;
5. design a skyline configuration where the result of a change in one assumption or situation can be displayed easily;
6. describe an alternative to the current standard in Central Europe of computing cable yarder properties in the field.
7. that is able to applicate in an open-source based software, with that a user could design and calculate standing skyline properties in a rapid and powerful way.

With this approach, the computation of a particular cable configuration requires solving a system of several non-linear equations. The solution to the equation system is numerical when the catenary is used and the solution is analytical when the parabola is used.

In order to show the validity of our new method, comparisons for basic configurations/problems with well-established computation approaches are presented. Further, to show the added value of our approach, we will show some more sophisticated configurations for which reference approaches for comparison are missing, as no appropriate methods were developed yet.

In order to show the validity of our new method, comparisons for basic configurations/problems with well-established computation approaches are presented. Further, to show the added value of our approach, we will show some more sophisticated configurations for which reference approaches for comparison are missing, as no appropriate methods were developed yet. We expect that the model behaviour of our approach is similar to the approach [14] for basic settings. However, our approach has the advantage that different configurations or settings can easily be considered by adding or removing additional equations to the equation system. The use of such non-linear analytical catenary equation systems to describe cable configuration is not new. It is used in other disciplines than forestry, for example to compute the initial equilibrium of railway overheads [18] or in civil engineering [19]. But however none of these approaches is tailored to describe the behavior of forestry cable yarding systems.

## 2. Background

Analytical assumptions used to predict skyline properties appeared in the 1900s. Initially, the catenary was identified as a shape representative of sagging cables (e.g. [11,20] or [15]). After some basic formulae were proposed, the parabola was used instead because the catenary was difficult to calculate.

[21] presented simplified formulae to compute the properties of a cableway by using a parabola. He analysed an unloaded span tensioned only by self-weight and loaded with a single or multiple load. At that time, cable skylines were only fixed to an anchor on one side. Because of the lack of hydraulic power or other techniques to tension the loose end, a tension weight was added to ensure that the tension of the unloaded and loaded cable was the same. However, this led to changing dimensions of sag. Further, elasticity and thermal expansion of the skyline do not influence such configurations. [20] and [22] further developed the equations introduced by [21] and proposed complementary formulae. The solutions remained approximations because of simplifications such as using one-sided fixed cables or weightless cables or plotting the self-weight against the chord instead of the catenary. Even though [13] only mentioned the pre-existing formulae in his definitive book about cable yarding, they become known in the field as the “Pestal formulae”. [13] recommended using ratios of maximum load to maximum tension of 1:5 to 1:10. Even today, the Pestal formulae are commonly used for approximate calculations of both-sided fixed-span cable yarders. The use of these formulae is acceptable for the calculation of the sag of an unloaded span, for which the variables relative weight per metre and mounting tension are known. On the contrary, the Pestal formulae produce inaccurate calculations for the loaded skyline. Under such conditions only the weight of the load is known; the tension and the sag of the loaded cable, resulting from the loaded equilibrium, are unknown. The following field procedure is standard: the proportion of the current load to the (unknown) current tension is set arbitrarily (in most cases 1:7), ignoring any relationship to the mounting tension and not considering whether the weight of the load is the maximum log load. With the help of this proportion, it may appear possible to calculate the corresponding sag and to prove the security of the cable yarder system, but this is not the case. Nonetheless, several project planning software packages currently in use in Europe are based on this analytical approach [23]. This procedure may lead to overloading of the skyline or ground-touching of the carriage. [24] described the calculation of an unloaded cable with use of the catenary in his definitive book about wire ropes, but he likewise calculated the loaded cable with only the aid of the parabola.

[25] described an approach for both-sided fixed spans for the first time and recognized that the elasticity (and with it the length of the cable) and the influence of temperature are significant in calculating the cable tension. As a result, he replaced the sagging cables with polygonal lines to enable computation. [11] used the fundamental relationship found by [25] to create a correlation between an unloaded both-sided fixed parabola and a loaded one. [12] and [15] improved this method. [12] used a Taylor series approximation instead of a catenary, and his numerical approach is therefore called “close-to-catenary”. He also presented solutions for different cases including multi-span effects, such as movement of the skyline over supports. Nevertheless, his formulae analysed just two situations: the loaded situation at the midspan position and the unloaded situation at one end. He calculated the sag based on approximations and as a function of former calculated simplified parameters. The approaches of [12] and [15] were the first to include a computation for both-sided fixed and multi-spanned catenaries that considered the influence of elasticity and the mounting tension.

[26] described an iterative process to calculate the maximum payload of a single-span catenary upon acceptance of a known sag via a system of equations, excluding the influence of elasticity and the mounting tension of the skyline (cable tensioned only by self-weight), by approximate catenaries from their chords. They added another iterative algorithm [27] to approximate the sag depending on the gross payload. [28] described a solution to analyse multisegmented catenary configurations based on iterative algorithms–the Newton method, the secant method and the “rigid Link” approach excluding the influence of elasticity or mounting tension. [29,30] and [16] developed tools to predict the payload capacity based on the formulae of [27], but included the elasticity of the skyline. The standard and popular cable planning software in North America, Skyline XL [31], formerly Logger PC 4.0, is probably based on this approach. However, we could not find its formulae and its foundations remain unclear.

[32] analysed the energy relationships of a suspended cable. With the help of linearized expressions, it was possible to calculate the load path, the gravitational potential energy and the elastic strain energy of extension when a point load or a distributed load was known. In his work “Cable Structures”, [14] compiled the theoretical correlation of an unloaded and a loaded single-span catenary, presenting formulae similar to those proposed by [25]. With his approach, however, a system of transcendental equations using a curvilinear abscissa in Lagrangian coordinates made it possible to calculate the abscissa of the sag of the loaded span or rather the longitudinal deflection of the sagging carriage spans for the first time. The involvement of a mounting tension or the influence of multi-span, however, was missing. Modelling Central European standing skyline configurations requires additional equations in the equation system, what is quite difficult because transformations of coordinates is required. Therefore, although the catenary is well represented, the application of this approach is limited.

[33] adapted Irvine’s approach and applied it to standing skyline specifications. As an initial parameter, they iteratively calculated the unstretched length of a both-sided fixed span (which is the same for the unloaded and loaded situations) with the help of the mounting tension, which does not correlate with the unstretched length. With that the approach also fails to involve the mounting tension to the approach of [14]. The software Cablehelp [34] is based on this approach.

The approach [14] is the only one to provide an unrestricted catenary solution, but it is difficult to extend and adapt to further applications because of the coordinate transformations required. To adapt it to Central European cable yarding configurations, the influence of the mounting tension should be included and multi-span layouts should be taken into account. The approaches introduced by [12] and [15] deal with these aspects, but they are based on simplifications to solve standard problems and ignore essential physical effects.

In this paper, we present a method to compute properties of loaded multi-span skylines that overcomes the shortcomings of the approaches discussed above. We first introduce the method, then make a comparison with established approaches and present the advantages of our approach for a few particular situations.

## 3. Materials and methods

### 3.1. Main assumptions

The main assumptions of our approach are:

1. The unstrained length of the both-sided fixed skyline (including the guylines) is constant. It is the link between the loaded and the unloaded situation.
2. The length of the skyline is recoverable in the linear elastic range, based on Hooke’s Law.
3. The shape of a sagging cable is a catenary. It is possible to substitute the catenary with a parabola, but this alternative is not fully included in the following equations.
4. An analysed static situation is represented by a system of equations that represent the equilibrium of forces/moments. The number of equations is equal to the number of unknown variables.

Certain parts of an equation that represent a physical effect, e.g. the inclination-dependent prolongation of the cable, can be considered or ignored. The presented system of equations is thus a flexible framework that can be used to consider individual configurations or particularities and is suitable for a wide range of applications.

### 3.2. Mathematical model

**Fig 1:**
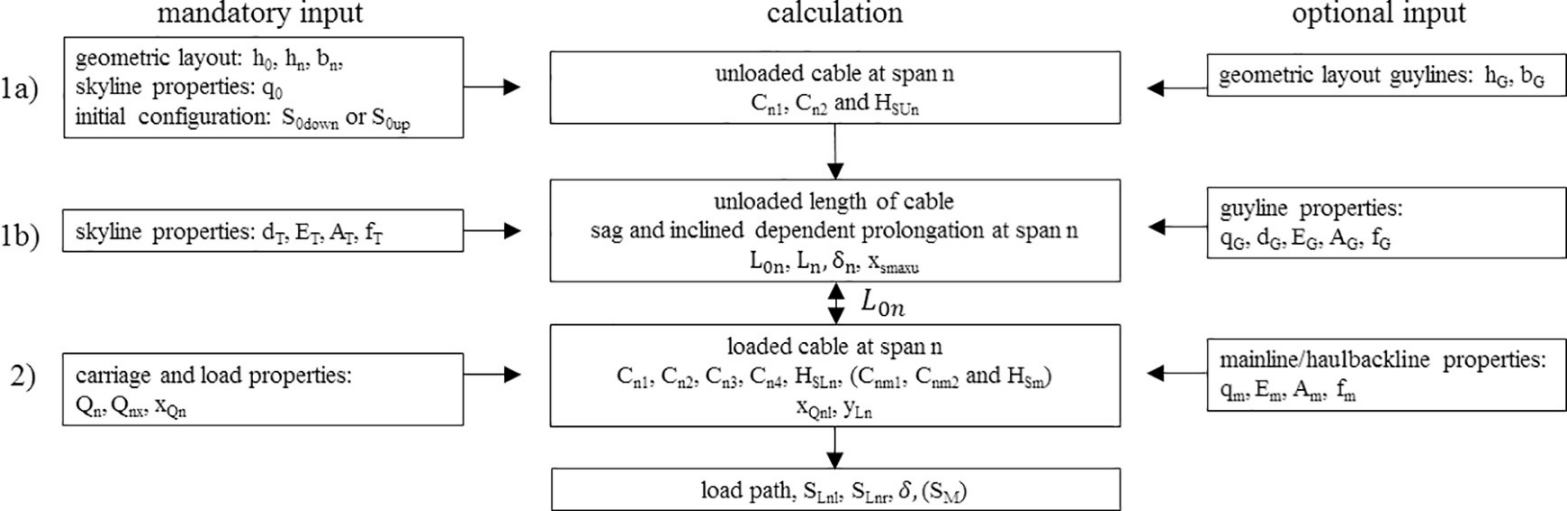
Calculation scheme. 1a) Calculation of the unloaded skyline span properties for all n spans. 1b) Calculation of the unloaded skyline properties. 2) Calculation of the skyline properties for a loaded cable at span n.

The connecting element of different load cases of both-sided fixed-anchor standing skyline configurations is the unchanging unstretched length (at reference temperature). Two load cases must be combined to calculate any properties of this configuration; therefore, one equation system for each load case is needed to produce a solution (for the calculation scheme see Fig 1).

The mathematical shape of a skyline is the catenary, a curve that a hanging cable assumes under self-weight when it is supported only at its ends without rotation restraints. Alternatively, one could use a parabola (formulae may be requested from the authors).

The equation of a catenary in Cartesian coordinates has the form

$$f_{n}(x,C_{n1},C_{n2},H_{Sn})=\frac{H_{Sn}}{q_{0}}*\cosh\left[\frac{q_{0}}{H_{Sn}}*(x+C_{n2})\right]+C_{n1}$$ (1)

where cosh is the hyperbolic cosine function and H_Sn_ is the horizontal component of the skyline tensile force at span n, which is independent from vertical acting forces and thus constant within one span. q_0_ is the self-weight per unit length of the skyline. Changing the term $\frac{H_{Sn}}{q_{0}}$ is equivalent to a uniform scaling of the catenary, as the term is the curvature radius at the angular point. The constant of integration C_n2_ is the displacement in the direction of the X-axis. C_n1_ is the displacement in the direction of the Y-axis.

**Fig 2:**
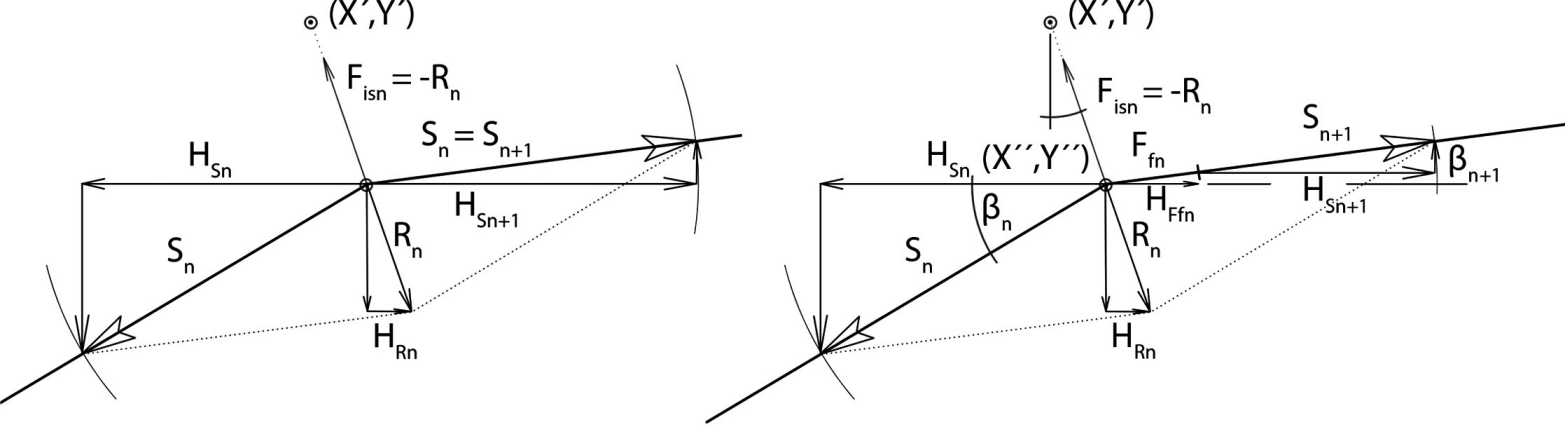
Force diagram at any break of slope (left) and at an intermediate support (right).

For multi-span catenary assemblies, the horizontal component of the skyline tensile force changes from one span to another, depending of the slope of their chords. Hence,

$$H_{Sn}+H_{Rn}=H_{Sn+1}(+H_{Ffn})$$ (2)

where H_Rn_ is the horizontal component of the resultant tensile force at any break of slope between span n and span n+1, and H_Sn+1_ is the horizontal component of the skyline tensile force at span n+1 (Fig 2). At an intermediate support the friction force F_fn_ appears when the tension in one span changes because of a changing load or a changing position of the carriage. Then, the cable likes to slip from an unloaded span (span n+1 in Fig 2; right) into a loaded span (span n in Fig 2; left). The horizontal part of the friction force (*H*_*Ffn*_) reduces H_Sn+1_ (or H_Sn_) (see Eqs 3 to 5).

With the help of

$$\frac{H_{Ffn}}{\cos\beta_{n+1}}={Ff}_{n}=\mu *R_{n}$$ (3)

and

$$R_{n}^{2}=S_{n}^{2}+S_{n}^{2}-(2*S_{n}*S_{n}*\cos\left(\beta_{n}-\beta_{n+1})\right)$$ (4)

it will be possible to calculate

$$H_{Ffn}=\mu *\sqrt{(2S_{n}^{2})-(2S_{n}^{2}*\cos\left(\beta_{n}-\beta_{n+1})\right)}*\cos\beta_{n+1}.$$ (5)

If (X´,Y´) is the point where the cable is rigged onto the intermediate support (with its pivot in X´´,Y´´), it is possible to identify the point of break of slope, which is dependent on the direction R_n_ of the carriage moving along a span. This step is possible with the help of a transformation of coordinates such that (X´,Y´) changes to (0,0) and (0,-L_isn_) is the pivot point of the intermediate support, where L_isn_ is the length of the tree strap to the intermediate support and μ is the static or dynamic coefficient of friction (e.g. Steel-Steel; μ is around 0.2).

Through rotation of coordinates,

$$X´´=X´*\cos\left(\beta_{n}-\beta_{n+1}\right)+Y´*\sin\left(\beta_{n}-\beta_{n+1}\right)$$ (6)

$$Y´´=-X´*\sin\left(\beta_{n}-\beta_{n+1}\right)+Y´*\cos\left(\beta_{n}-\beta_{n+1}\right)$$ (7)

(X´´,Y´´) could be used as changing mounting points to compute the spans.

**Fig 3:**
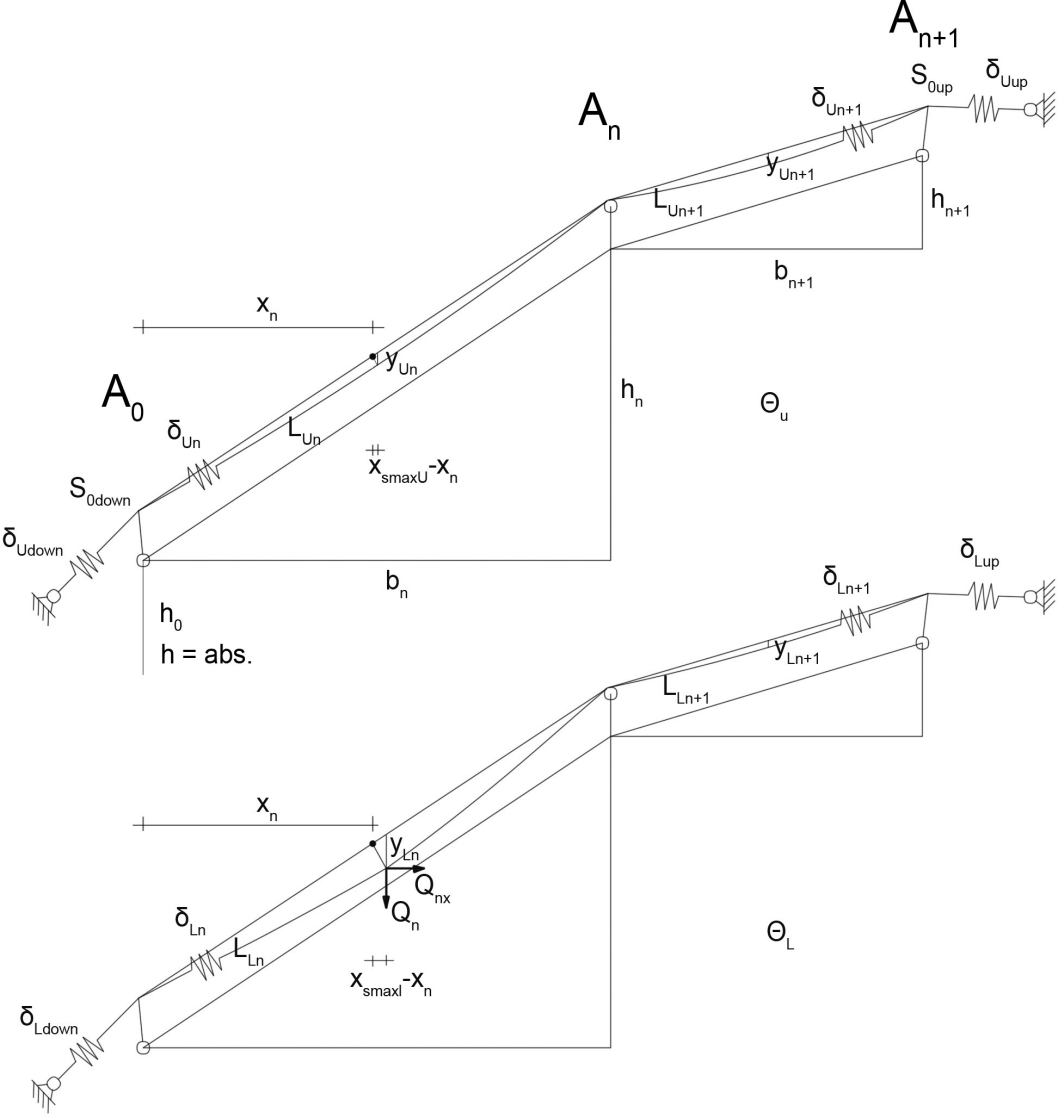
Definition of geometric parameters.

With Central European cable yarding, the skyline is fixed at the mobile tower and the remote anchor. Between these fixed points, the skyline is held up by skyline supports (so that the carriage is able to pass) or spars (so that the carriage is unable to pass). The entire unstretched length L_0_ of the skyline is invariable, but slanting of the mobile tower is possible because of elasticity of the guylines. At the location of the mobile tower, the mounting tensile force S_0_ appears (visualized as S_0up_ or S_0down_ in Fig 3).

Fig 3 shows the geometric layout of an unloaded (top, index ‘U’) and loaded (down, index ‘L’) multi-span with both-sided guylines (carriage clamped). For simplification, additional needed cables or swinging intermediate supports are not displayed in the figure. We assume that the skyline is fixed anchored to the mobile tower and to anchor trees and that the guylines are elastic. This can change the dimensions of the first and last span. All symbols are defined in the list at the end of the paper.

### 3.3. Computation of the unloaded cable

In the first step, the unloaded cable is analysed. The boundary conditions at span n are

$$h_{n-1}=f_{n}(0,C_{n1},C_{n2},H_{SUn})$$ (8)

left mount span n, where *H*_*SUn*_ is the horizontal component of the skyline tensile force at the unloaded span n;

$$h_{n}=f_{n}(b_{n},C_{n1},C_{n2},H_{SUn})$$ (9)

right mount span n;

$$\frac{S_{0}}{H_{SUn}}=cosh(C_{n1}*\frac{q_{0}}{H_{SUn}})$$ (10)

functional relationship of S_0_ and H_SU_, if the mounting tensile force S_0_ is located at h_0_, or

$$\frac{S_{0}}{H_{SUn}}=cosh((b_{n}+C_{n1})*\frac{q_{0}}{H_{SUn}})$$ (11)

functional relationship of S_0_ and H_SU_, if the mounting tensile force S_0_ is located at h_n_;

$$\frac{S_{n-1}}{H_{SUn}}=cosh((b_{n}+C_{n1})*\frac{q_{0}}{H_{SUn}})$$ (12)

general functional relationship of S_n-1_ and H_Sun_

$$S_{n-1}=S_{n}-q_{0}(h_{n+1}-h_{n})$$ (13)

relationship between tensile forces at different elevation.

The system of non-linear equations for each span consists of Eqs (8) and (9) and one Eqs from (10) to (12), resulting in three equations for each span, and it also contains three unknown variables (*C*_*n*1_, *C*_*n*2_, *H*_*SUn*_). It was implemented in Python version 3.7 [35] and solved using “fsolve” from the package “SciPy.optimize”. The solver “fsolve” finds the roots of a function. It returns the roots of a non-linear equation system defined by func(x) = 0 when given a starting estimate. “fsolve” is based on the MINPACK subroutine HYBRD that was developed by [36]. The purpose of HYBRD is to find a zero of a system of non-linear functions with the same dimension of variables by modifying the Powell hybrid method. As a result, “fsolve” determines the integration constants C_n1_, C_n2_ and H_SUn_ for each span. One can use any other appropriate solver than “fsolve”, too.

The unstretched length of every single span is then calculated with

$$L_{0n}=L_{n}-\delta_{n}$$ (14)

where

$$L_{n}=\int_{b_{n-1}}^{b_{n}}\sqrt{1+{\left(\frac{d}{dx}f_{n}\right)}^{2}}dx$$ (15)

is the stretched length of the skyline.

$$\delta_{n}(H_{SUn})=\frac{H_{SUn}}{E_{S}*A_{S}*f_{T}}*\left[\int_{b_{n-1}}^{b_{n}}[{\left[\frac{(h_{n}-h_{n-1})*q_{0}}{2*H_{SUn}*sinh(\frac{(b_{n}-b_{n-1})*q_{0}}{2*H_{SUn}})}\right]}^{2}+1]dx\right]$$ (16)

is the inclination-dependent elastic prolongation of the skyline, where E_S_ is the Young-modulus of the skyline, A_S_ is the cable’s metallic cross-sectional area, and f_T_ is the fill factor of the cable. The equation was derived with help of the relationships of hyperbolic functions in [37] (cf. derivation of the formula in the Appendix A). That means that the elastic prolongation of a sagging cable depends on the tension and the inclination of several parts of the cable. The general elastic prolongation at strongly inclined sections is larger than at flat ones.

$$L_{0}=\sum_{i=1}^{n}L_{0n}$$ (17)

is the entire unstretched length of the skyline.

The location *x*_*smaxu*_ of the maximum sag of span n (see (18)) is calculated with:

$$\frac{\left(h_{n}-h_{n-1}\right)}{b_{n}}=\frac{d}{dx}f_{n}(x_{smaxu},C_{n1},C_{n2},H_{Sn}),$$ (18)

so that the maximum sag is

$$y_{Un}(x_{smaxu})=\frac{\left(h_{n}-h_{n-1}\right)}{b_{n}}*x_{smaxu}+\frac{\sum_{i=1}^{n}\left(b_{n}*h_{n-1}-h_{n}*b_{n-1}\right)}{b_{n}}-f_{n}(x_{smaxu})$$ (19)

If the skyline ends directly at an anchor, the part of skyline between anchor and spar can be computed as a separate span. If the skyline ends at the tower yarder, a number n_G_ of guylines with different elastic behaviours anchor the tower itself. We assume that the following variables of every guyline are known by measurement in the field: the slanted distance from the bottom of the tower to the anchor and its slope, both projected on the plane of the skyline (L_Gn1_ and γ_Gn1_), the lateral distance from the plane of the skyline to the anchor L_Gn2_, and the height of the tower L_Gn3_. The length of the single guyline is therefore

$$L_{Gn}=\sqrt{{L_{Gn2}}^{2}+{\left((\cos\;\gamma_{Gn1}*L_{Gn1}\right)}^{2}+{\left(L_{Gn3}\pm \sin\;\gamma_{Gn1}*L_{Gn1}\right)}^{2})}$$ (20)

where + is used when *γ*_*Gn*1_ is < 0 and–is used when *γ*_*Gn*1_ > 0. Its angle γ_Gnl_ of lateral deflection from the plane of skyline is

$$\gamma_{Gnl}=\arctan\left(\frac{L_{Gn2}}{\sqrt{{\left(\cos\gamma_{Gn1}*L_{Gn1}\right)}^{2}+{\left(L_{Gn3}\pm \sin\gamma_{Gn1}*L_{Gn1}\right)}^{2}}}\right)$$ (21)

Every sidewarded guyline need another guyline on opposite site of the plane of skyline, that angle of lateral deflection is γ_Gnr_. Both angles (left and right the plane of skyline) will need to calculate the tension in every single guyline S_Gn_ by law of sines:

$$S_{Gn}=\frac{\sin\left(\gamma_{Gnl}\right)*S_{n}}{n_{G}*sin(180{}^{\circ}-\gamma_{Gnl}-\gamma_{Gnr})}$$ (22)

The unstretched length of a guyline is:

$$L_{Gn0}=L_{Gn}\left(1-\frac{S_{Gn}}{E_{G}*A_{G}}\right)$$ (23)

where E_G_ is the Young-modulus of the guyline and A_G_ is the cable’s metallic cross-sectional area (including the cable’s fill factor).

Now the static behaviour and all previously unknown variables (H_SUn_, C_n1_, C_n2_) of the unloaded skyline can be determined by a straight solution of the catenary. Thus, the unstretched length of the entire skyline and the guylines are the link between the unloaded and the loaded situation.

### 3.4. Computation of the loaded cable

In the second step, the loaded cable is analysed. Because of the lateral force of the carriage, the span will be divided into two single catenary segments. The boundary conditions of span n are

$$h_{n-1}=f_{n1}(0,C_{n1},C_{n2},H_{SLn})$$ (24)

left mount span n, where *H*_*SLn*_ is the horizontal component of the skyline tensile force at the loaded span n;

$$h_{n}=f_{n2}(b_{n},C_{n3},C_{n4},H_{SLn})$$ (25)

right mount span n;

$$f_{n1}(x_{Qnl},C_{n1},C_{n2},H_{SLn})=f_{n2}(x_{Qnl},C_{n3},C_{n4},H_{SLn})$$ (26)

connection in carriage at span n, where *x*_*Qnl*_ is the horizontal location of the longitudinal deflection of the sagging carriage;

$$H_{SLn}*\left(\frac{d}{dx_{Qnl}}f_{n1}\left(x_{Qnl},C_{n1},C_{n2},H_{SLn}\right)\right)+Q_{n}=H_{SLn}*\left(\frac{d}{dx_{Qnl}}f_{n2}\left(x_{Qnl},C_{n3},C_{n4},H_{SLn}\right)\right)$$ (27)

equilibrium of vertical forces at span n, where the carriage is located at an unknown horizontal position x_Qnl_ and Q_n_ is the weight of the carriage and the additional load in span n.

Furthermore, Eq (28) ensures that the cable length of the loaded cable, calculated from different sources, is consistent. On the left side of the equation, the loaded cable length is derived from the entire unstretched cable length, its loaded elastic prolongation and the elastic prolongation of the guylines. In this formulation the influence of the elastic prolongation of the guyline on the entire cable length is considered, but the displacement of tower tips is disregarded because it does not have a significant effect. One can consider this effect with the help of Eqs (5) to (7) (like a swinging intermediate support). The right part of the equation calculates the loaded cable length based on the geometry of the individual segments of the loaded catenary.

$$\sum_{i=1}^{n}L_{0n}+\sum_{i=1}^{n}\delta_{n}(H_{SLn})\pm \sum_{i=1}^{n}L_{0n}\varepsilon \Delta t+{\sum L}_{Gn0}(1+\frac{S_{Gn}*L_{Gn}}{*E_{G}*A_{G}})=\sum_{i=1}^{n}\left[\int_{b_{n}-1}^{x_{Qnl}}\sqrt{1+{\left(\frac{d}{dx}f_{n1}(x_{Qnl},C_{n1},C_{n2},H_{SLn})\right)}^{2}}dx_{Qnl}+\int_{x_{Qnl}}^{b_{n}}\sqrt{1+{\left(\frac{d}{dx}f_{n2}(x_{Qnl},C_{n3},C_{n4},H_{SLn})\right)}^{2}}dx_{Qnl}\right]$$ (28)

where ε is the coefficient of thermal expansion, Δt is the increase (+) or decrease (–) in temperature from Θ_u_ (the temperature of the unloaded situation.

$$\frac{\int_{0}^{x_{Qnl}}\sqrt{1+{\left(\frac{d}{dx}f_{n1}(x_{n},C_{n1},C_{n2},H_{SLn})\right)}^{2}}dx_{n}}{\int_{x_{Qnl}}^{b_{n}}\sqrt{1+{\left(\frac{d}{dx}f_{n2}(x_{n},C_{n3},C_{n4},H_{SLn})\right)}^{2}}dx_{n}}=\frac{x_{Qn}}{b_{n}-x_{Qn}}$$ (29)

describes the location of the carriage alongside the span (Eq 29), where x_Qn_ represents the horizontal position of the carriage as an arbitrary x-coordinate in span n (*b*_*n*−1_ < *x*_*Qn*_ < *b*_*n*_). The load path is the solution of x_Qnl_, where x_Qn_ is a control variable running from the left to the right side of every span.

$$0=\left[\frac{H_{SLn}}{\cos\propto_{n}}+q_{0}*\left[h_{n}-(\frac{h_{n}-h_{n-1}}{2}\right)]\right]-\left[\frac{H_{SLn+1}}{\cos\propto_{n+1}}-q_{0}*[h_{n+1}-(\frac{h_{n+1}-h_{n}}{2})]-\frac{H_{Frn}}{\cos\propto_{n+1}}\right]$$ (30)

is the relationship between different horizontal parts of the loaded tensile force of cable for each span n (see Eq 30), including the friction force F_rn_, when an intermediate support is used. α_n_ is the slope of the chord in span n ($\alpha_{n}=arctan(\frac{h_{n}}{b_{n}})$).

Using “fsolve” again, the solution of this system of non-linear equations is computed numerically (Eqs 24 to 30). By using the parameters of catenaries C_n1_, C_n2_, C_n3_ and C_n4_, the resulting H_SLn_ and the horizontal location of the longitudinally deflected carriage x_Qnl_ can be calculated. The maximum sag y_Ln_ (Eq 19), the cable tensile force left and right of the carriage (Eq 12) and the graphical presentation of all spans can then be calculated based on the location of all carriages.

If the catenary is described by a parabola, an analytical solution for the system of linear system of equations is possible with our approach.

Using the Eq 31,

$$H_{SLnl}\pm Q_{nx}=H_{SLnr}$$ (31)

the impact of slanted forces can be considered. This will be needful during the transversal cable-pull of logs when the carriage is clamped onto the skyline. Q_nx_ is the horizontal component of the resulting force Q_r_, where the indices left (l) and right (r) indicate the position relative to Q_r_ (H_SLnl_ and H_SLnr_ are unknown and need this additional Eq 31 in order to solve the system of equations). +Q_nx_ is used if the force is oriented to the left and -Q_nx_ if the force is oriented to the right.

The following example shows situations in which a change in the formula structure is necessary. Any situation can be simulate by adding and adapting the formulas describing the state of equilibrium.

So far, we have considered the case where the carriage is clamped at the skyline without the influence of any mainline. In the following we introduce the case where the carriage is held by a mainline, but still clamped. When the influence of additional cables (mainline) is considered, Eq 31 is replaced by Eq 32, and Eqs 33 to 35 are introduced.

$$H_{SLnl}\pm Q_{nx}=H_{SLnr}+H_{m}$$ (32)

$$f_{n1}(x_{Qnl},C_{n1},C_{n2},H_{SLn})=f_{m}(x_{Qnl},C_{nm1},C_{nm2},H_{m})$$ (33)

left mount in carriage

$$h_{n}=f_{m}(b_{n},C_{nm1},C_{nm2},H_{m})$$ (34)

right mount of mainline

$$\frac{S_{m}}{H_{m}}=cosh((b_{n}+C_{nm1})*\frac{q_{m}}{H_{m}})$$ (35)

functional relationship of S_m_ and H_m_, if the mounting tensile force of the mainline S_m_ is located at h_n_. An additional cable works like an unloaded cable (Eqs 8, 9 and 12, that are combined with the help of the Eq (32). Eq (35) represents the functional relationship of S_m_ and H_m_, if the mounting tensile force of the mainline S_m_ is located at h_n._

Furthermore, Eq 27 is changed to

$$H_{SLn}*\left(\frac{d}{dx_{Qnl}}f_{n1}\left(x_{Qnl},C_{n1},C_{n2},H_{SLn}\right)\right)+Q_{n}=H_{SLn}*\left(\frac{d}{dx_{Qnl}}f_{n2}\left(x_{Qnl},C_{n3},C_{n4},H_{SLn}\right)\right)\pm \sqrt{{\left(f_{m}(x_{Qnl},C_{nm1},C_{nm2},H_{m})\right)}^{2}+{H_{m}}^{2}}$$ (36)

(+ cable is oriented upwards;–cable is oriented downwards).

In the following we introduce the case where the carriage is held by the mainline, but not clamped on the skyline. As long as Eq 29 is used for computation, the carriage is clamped at the skyline. Declamping the carriage means that Eqs 29 and 35 are ignored, and the length of the mainline is therefore fixed as

$$L_{m}=\int_{x_{Qnl}}^{b_{n}}\sqrt{1+{\left(\frac{d}{dx}f_{m}\right)}^{2}}dx$$ (37)

where L_m_ is calculated as if the carriage were clamped. Furthermore, an additional Eq 38 is needed to describe that the tension of the mainline is now equal to the downhill-slope force of the carriage.


$$H_{m}=Q_{n}*\frac{\sin(\tan^{-1}(\frac{d}{dx_{Qnl}}f_{n1}(x_{Qnl},C_{n1},C_{n2},H_{SLn}))+\tan^{-1}(\frac{d}{dx_{Qnl}}f_{n2}(x_{Qnl},C_{n3},C_{n4},H_{SLn}))}{2}$$ (38)

Then the carriage moves downhill alongside the skyline until the mainline is tensioned. If Q_n_ has changed, the position of the carriage is not the same as in the preclamped condition.

## 4. Results

**Fig 4:**
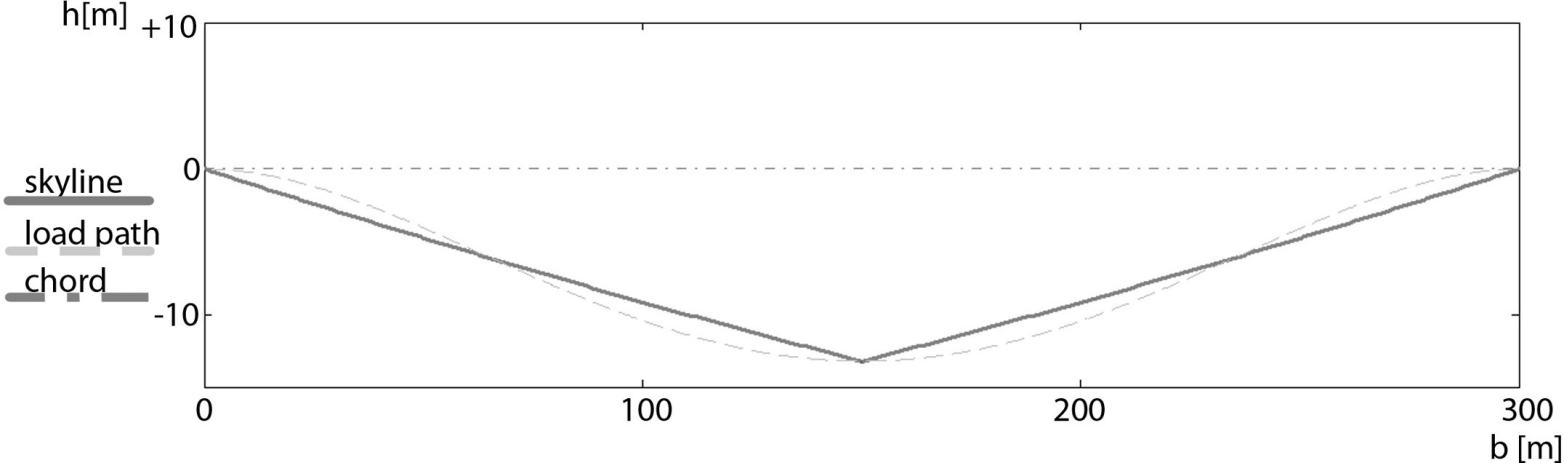
Sag with carriage at midspan position in a flat single-span configuration.

**Fig 5:**
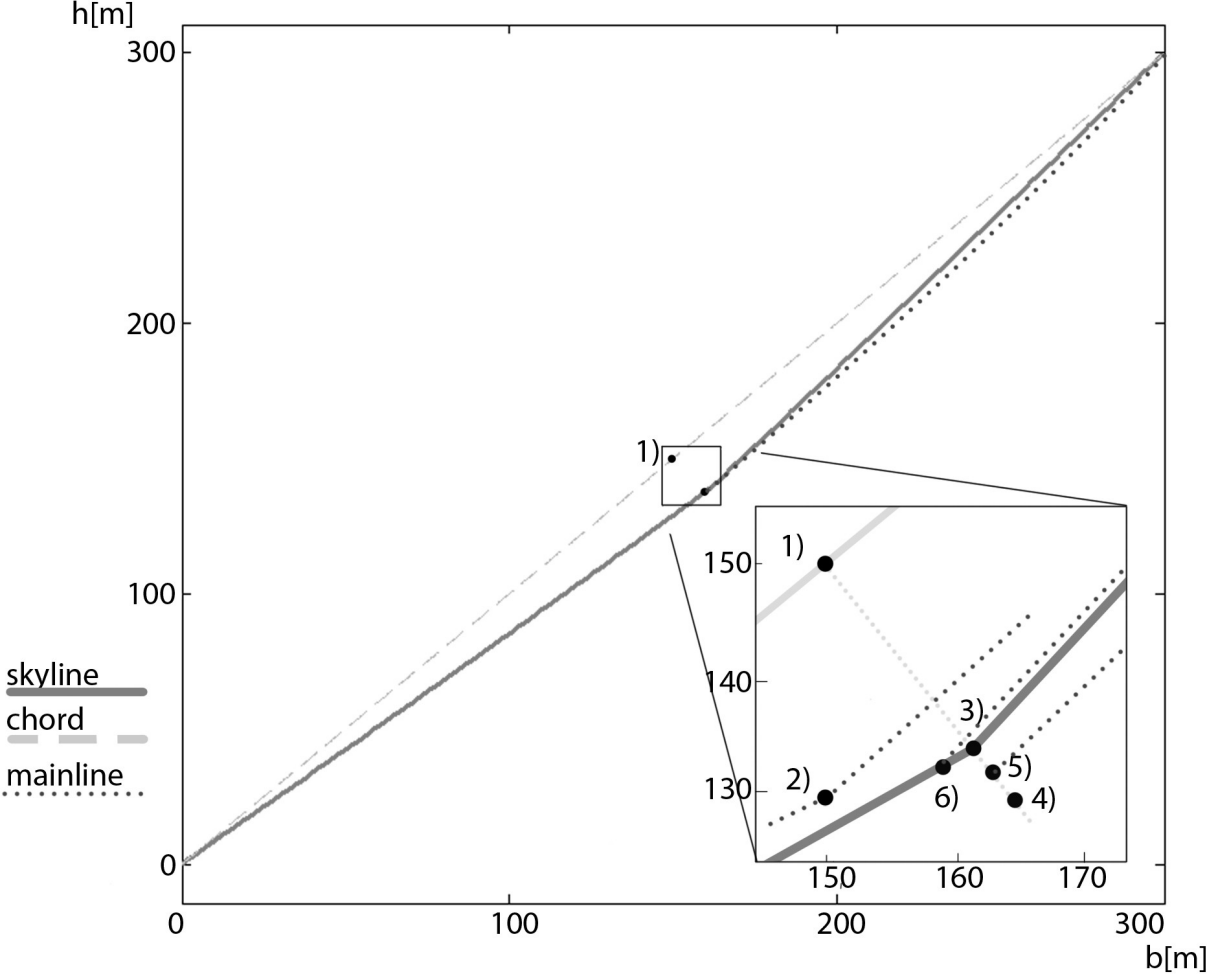
Sag with carriage at midspan position in an inclined single-span configuration.

In order to show the validity of our new method, comparisons for basic configurations/problems with well-established computation approaches are presented. Further, to show the added value of our approach, we will show some more sophisticated configurations for which reference approaches for comparison are missing, as no appropriate methods were developed yet. We compare the performance and limitations of our approach with those of the approaches proposed by [12–15] with flat and (in the next step) inclined examples with a single load in the middle of the span (see Tables 2 and 3 for specific values and Figs 4 and 5 for their graphical display). To get comparable results, we refined the theory of [14] to include the impact of mounting tension. However, because this approach is not easy to adapt to multi-span configurations, our comparison example is a single-span one.

**Table 2:** Display of the solutions of a flat example.

Variable	Our approach	Pestal (1961) [13]	Zweifel (1960) [12]	Czitary (1961) [15]	Irvine (1981) [14]
y unloaded in m	3.297	3.296	3.293	3.293	3.287
L unloaded in m	300.097	300.097	300.096	300.096	300.086
L unstretched in m	299.152		300.151	300.151	299.141
H_SL_ in kN	127.0		127.4	129.4	126.901
y loaded in m	13.143	27.818	13.102	12.904	13.147
L loaded in m	301.149		301.159	301.19	301.147
x_Qnl_ in m	150	150	150	150	150

flat example with b = 300 m, **h = 0 m**, E_S_ = 100 kN/mm^2^, Q = 2000 kg*9.81 m/s^2^, q_0_ = 17.564 N/m (diameter 20 mm), f_T_ = 0,606, S_0_ = 60 kN (located at h_0_).

**Table 3:** Display of the solutions of an flat example.

Variable	Our approach	Pestal (1961) [13]	Zweifel (1960) [12]	Czitary (1961) [15]	Irvine (1981) [14]
y unloaded in m	6.319	6.587	6.587	6.587	6.298
L unloaded in m	424.39	423.67	424.40	424.40	423.98
L unstretched in m	423.167		423.005	423.005	422.998
H_SL_ in kN*	77.45		77.40	76.00	78.04
y loaded in m*	22.460	29.182	22.637	21.948	22.465
L loaded in m	425.307		425.128	425.099	424.73
x_Qnl_ in m	161.244	150	150	150	161.121

Table 3 inclined example with b = 300 m, **h = 300 m**, E_S_ = 100 kN/mm^2^, Q = 2000 kg*9.81 m/s^2^, q_0_ = 17.564 N/m (diameter 20 mm), f_T_ = 0,606, S_0_ = 60 kN (located at h_0_); *at position of max. deflection.

1) midspan; 2) sagged location of a carriage clamped at midspan; 3) sagged location of a carriage clamped at midspan under the impact of an additional horizontal force from the right; 4) sagged location of a carriage clamped at midspan under the impact of an additional horizontal force from the right and a mainline from uphill; 5) unclamped carriage fixed on the mainline from uphill when the weight of the carriage is larger than under preclamped conditions.

**Fig 6:**
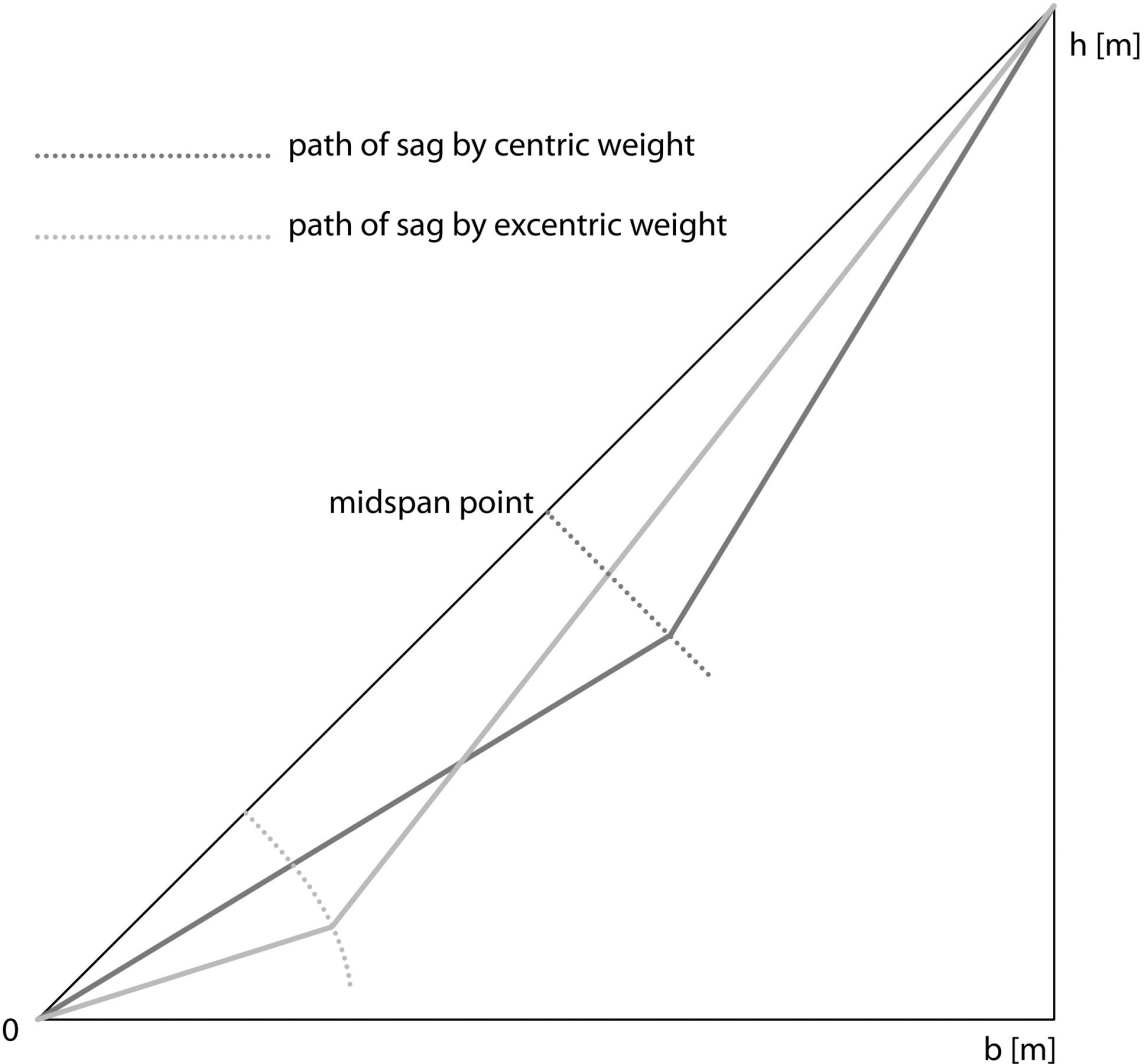
Path of a centric and excentric sagging carriage, due to an increasing load of the carriage.

Tables 2 and 3 show that our results are almost identical to those of [14], which also avoids specific simplifications. The method of [13] show similar results for the unloaded situation but as described before, the method overestimates the sag of the loaded situation. The methods of [12] and [15] also result in similar solutions, but for inclined spans the location of the maximum sag is different than in our approach and that of [14] (see Table 3 and point 2 versus point 3 in Fig 5). This discrepancy arises because the influence of longitudinal deflection means that the real position of the carriage differs from the expected position. In our example, the failure deviation is 11 m! In most of the analysed approaches it is assumed that the maximum sag is at midspan. Midspan is the position where a carriage has the largest dimension of lever arms for sagging. However, at that point the sag of a skyline is not affected in the direction of gravity, but rather rectangular to the chord. As a result, the maximum sag for inclined spans is shifted from the midspan (discrepancies from the exact rectangular direction can occur because of the inclination-dependent elastic prolongation of the skyline). The sag of a loaded skyline, if the carriage is not located at the midspan position, appears curved in the direction of the shorter cable, due to the increased load of the carriage and the larger elastic prolongation of the longer cable (Fig 6). Point 4 in Fig 5 shows the sagged location of a carriage clamped at midspan under the impact of an additional horizontal force from the right. The sag increases due to the additional load, but the direction is not influenced. Point 5 in Fig 5 shows the sagged location of a carriage clamped at midspan under the impact of an additional horizontal force from the right and a mainline from uphill. At upward direction, when the additional mainline affects the carriage, the location of the carriage is near to point 3 in Fig 5. If the weight of the carriage and its load increases while clamped and the unclamped carriage is now fixed only at the mainline from uphill, the location of the carriage is point 6 in Fig 5.

If the skylines showed in the examples in Table 3 are extended by 10 mm, the increasing of sag is around 48 mm. That means that the consideration of (inclination-dependent) elastic prolongation or longitudinal deflection is essential for predicting the behaviour of skylines or additional cables.

**Fig 7:**
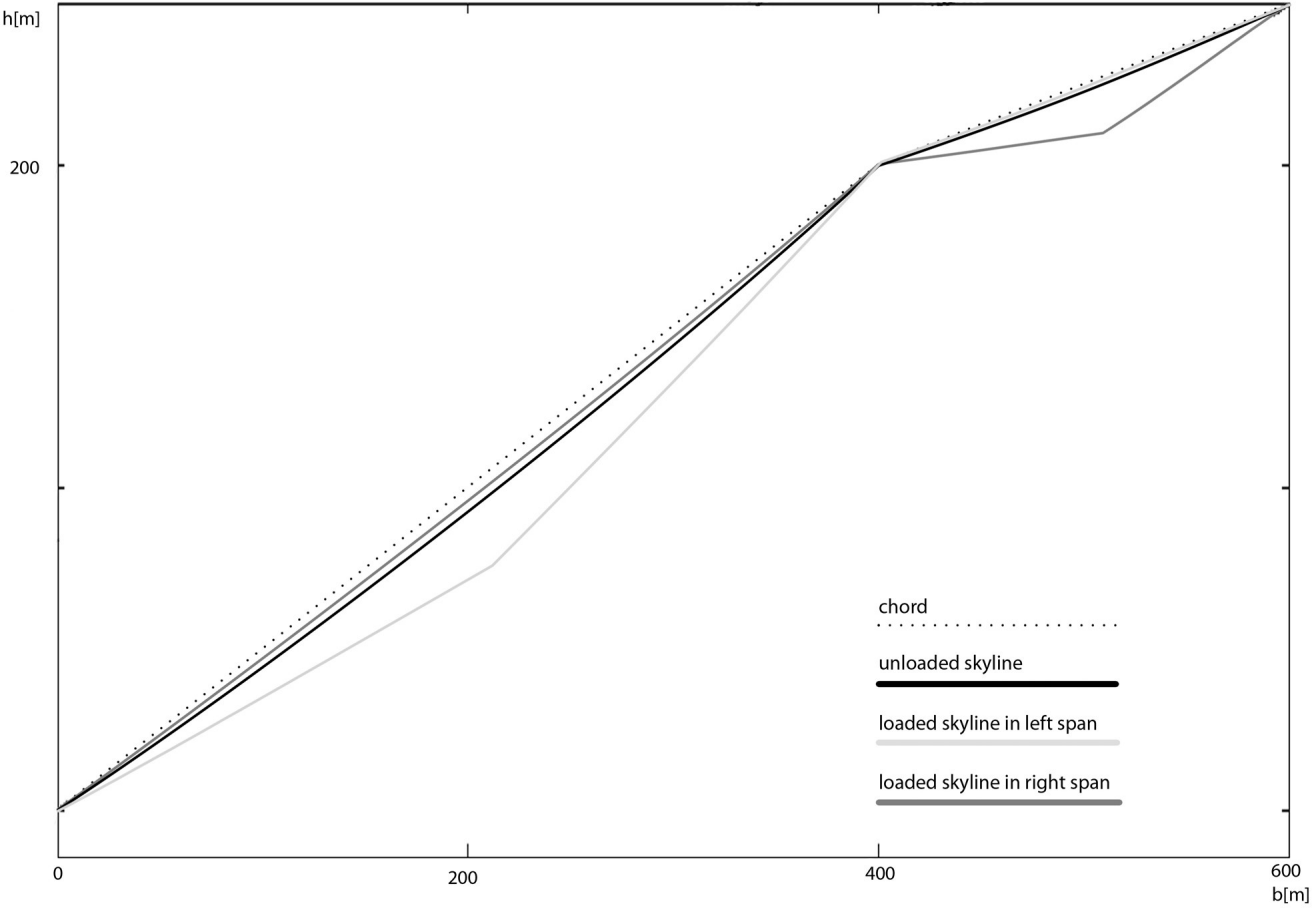
Cable layout with a double-span configuration.

When analysing our approach with an example of a double-span configuration, the interaction between the two parts of the skyline becomes apparent (Fig 7). The tension in both spans, the loaded as well as the unloaded one, increases. The sag of the unloaded span is less than in a situation, where the skyline is completely unloaded. With our approach it is possible to include more than one carriage in a span (for two carriages a span one need three catenary segments, Eqs (26) and (27) doubled and Eq (28) modified with an additional term for the third segment) or multiple carriages in multi-span configurations without any further formulae. The calculation of double-span configuration is as well possible with the methods of [12] and [15]. But both methods show the described inaccuracy at inclined spans. The methods of [13] and [14] are not suitable for multi-span configurations.

The unloaded cable situation is drawn in black color. The graph in light gray color represents the loaded skyline when the carriage is located at midspan of the left span, and the cable in the right span is more tensioned than in the unloaded span and its sag is smaller. The graph in dark gray color represents the opposite situation, where the carriage is located at midspan of the right span.

## 5. Discussion

The presented approach was compared with other considered approaches with the aid of a flat and an inclined example. The results of the flat example (Table 2) are almost identical meanwhile the results of the inclined example differs (Table 3). With the help of Table 1 the abilities and lacks of known methods were analysed. We identified, that the most methods except [14] does not consider longitudinal deflection and with that, they assume–especially at inclined spans–the point of maximum sag at a wrong direction or rather underestimate the load path. The method of [14] again is not suitable to calculate multi-span configurations or to integrate the influence of mounting tension. Our approach is a solution to compute both-sided fixed multi-span catenaries with moveable loads, including the influences of elasticity, temperature, friction, slanted single or multiple loads and mounting tension without any simplifications. In contrast to conventional approaches, it is possible to consider essential physical effects like the inclination-dependent elastic prolongation of the cable, elastic guylines or the longitudinal deflection of the sagging carriage. Only with the consideration of these essential physical effects, it will be possible to calculate typical Central European cable yarder configurations. With that, this approach could bridge a gap in computing these configurations with improved accuracy.

Because of the multiplicity of possible configurations, however, it is not possible to determine the accuracy of a value against one resulting from another approach without a wide range of field tests.

## 6. Conclusions

The basic intention of this paper is to present a new method of mathematical computation and to compare it with known methods. We showed that our results are very near to the most exact solution, but it can–in contrast with the other competing solutions—deal with the Central European cable yarder configurations or wider range of cases. That is the main value-add of our paper. The verification of theoretical results with real measurements of field tests will be a topic for a possible future paper.

The calculated values show that the stretched length of the cable determines the cable’s behaviour: its sag and tension, and the load path. The consideration of essential physical factors and the use of the catenary helps to form an image of the real cable situation. The study of the impact of small differences in corresponding variables, e.g. the stretched length of a cable compared with the sag, shows the importance of using calculations that are as exact as possible. Computing the parameter of a cable road with a simplified approach increases the risk of underrating the tension of the skyline or the load path of the carriage. Up to now, the methods of [12] and [15] are the best approaches available for Central European cable yarder configurations. They are useful for practical applications if ignoring of essential physical effects can be tolerate. The method of [14], which does not use simplifications but relies on coordinate transformation, which makes it difficult to use in applications beyond standard problems.

In our approach involving a system of non-linear equations, the “fsolve” algorithm converged in all tested cases to a realistic solution. Our approach provides a flexible framework for considering individual configurations or particularities: additional equations can be included in the equation system, and common simplifications, approximations or transformations of coordinates that are valid for only a defined range of locations can be avoided. Our approach also makes it possible to immediately differentiate between special cases in multi-span configurations or between locations along the cable.

In the future, we aim to extend our approach to dynamical computation relevant to typical cable logging situations [38] and plan to combine it with a spatially explicit optimization tool for cable layouts [9].

## 7. Appendix

A) Derivation of formula (16):

$$\delta =\int_{1}^{2}\frac{S*ds}{A*E}$$ (1A)

basic formula of the elastic prolongation of a curve (Eq 1A)


$$\frac{S}{H}=\frac{ds}{dx}$$ (2A)

**Fig 8:**
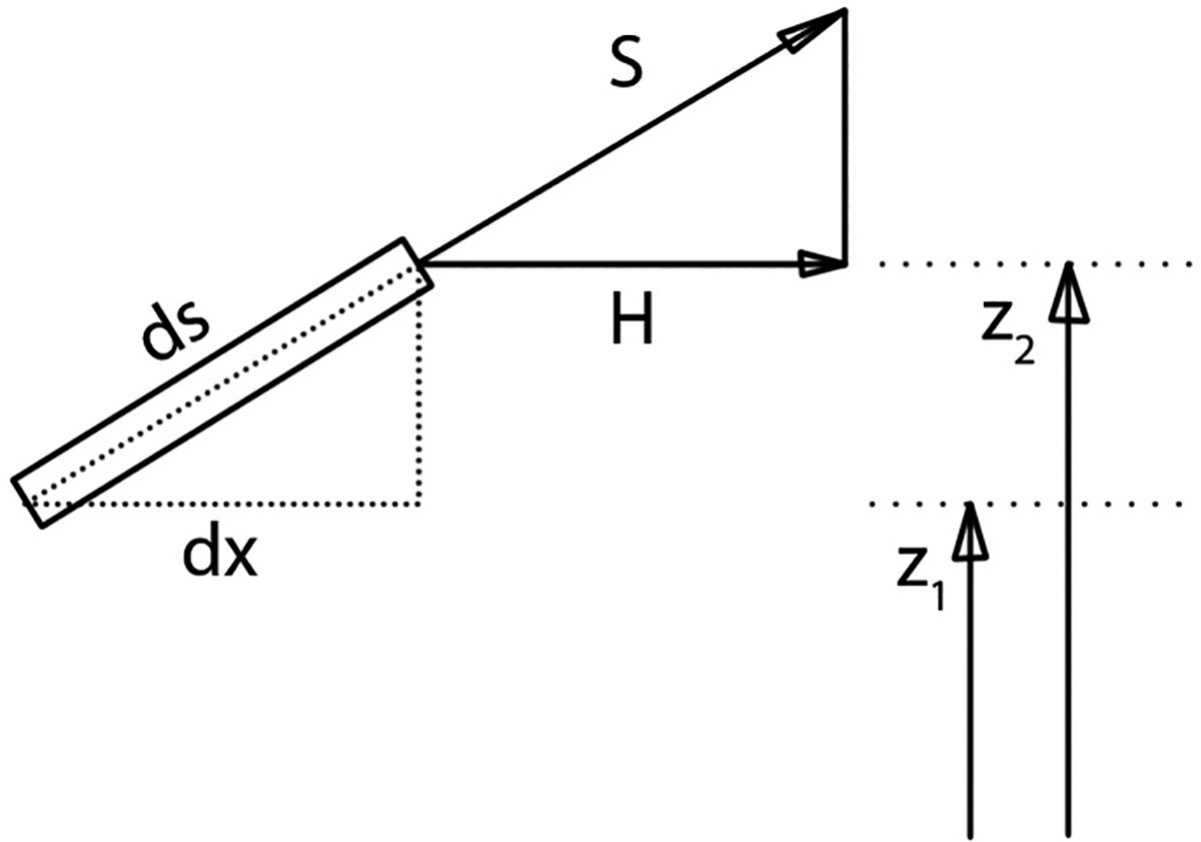
Display of an infinitesimal cable segment.


Eq 2A shows the context of Fig 8, with that

$$\delta =\frac{H}{A*E}\int_{1}^{2}\frac{ds}{dx}*ds=\frac{H}{A*E}\int_{1}^{2}{\left(\frac{ds}{dx}\right)}^{2}*dx$$ (3A)

$$L=\frac{H}{q}*\sinh\frac{q}{H}x$$ (4A)

arc length between x = 0 and x = x, (see Eqs 3A to 5A) with (Czichos & Henneke 2012) [37]:

$${\left(\frac{ds}{dx}\right)}^{2}={cosh}^{2}*\frac{q}{H}x+constant$$ (5A)

The vertical height of an element of sagging cable is (see Eq 6A):


$$z_{2}-z_{1}=\frac{H}{q}(\cosh\frac{q}{H}*(x+\frac{b}{2})-\cosh\frac{q}{H}*(x-\frac{b}{2})$$ (6A)

Again, with (Czichos & Henneke 2012) [37] (see. Eqs 7A to 9A):


$$z_{2}-z_{1}=\frac{2*H}{q}sinh\frac{q}{H}x*sinh\frac{b*q}{2*H}$$ (7A)

$$sinh\frac{q}{H}x=\frac{h*q}{2*H*sinh\frac{b*q}{2*H}}$$ (8A)

$$cosh\frac{q}{H}x=\sqrt{{\left(\frac{h*q}{2*H*sinh\frac{b*q}{2*H}}\right)}^{2}}+1$$ (9A)

3A, 5A and 9A will combine to 10A


$$\delta_{n}(H_{SUn})=\frac{H_{SUn}}{E_{S}*A_{S}*f_{T}}*\left[\int_{b_{n-1}}^{b_{n}}[{\left[\frac{(h_{n}-h_{n-1})*q_{0}}{2*H_{SUn}*sinh(\frac{(b_{n}-b_{n-1})*q_{0}}{2*H_{SUn}})}\right]}^{2}+1]dx\right]$$ (10A)

## Abbreviations

index U: unloaded
index L: loaded
index down: bottom end of cable road
index up: upper end of cable road
index l: left of carriage
index r: right of carriage
index s: skyline
index m: mainline
index g: guyline
index n: span
A_S_: skyline’s metallic cross-sectional area
A_G_: guyline’s metallic cross-sectional area (incl. fill factor)
b_n_: horizontal distance of span n
C_n1_: constant of integration of catenary equation at span n
C_n2_: constant of integration of catenary equation at span n
E_S_: Young-modulus of skyline
E_G_: Young-modulus of guyline
f_n_: catenary equation/skyline at span n
f_m_: catenary equation/mainline
f_T_: fill factor of skyline
h_0_: reference height
h_n_: elevation at right side of span n; at left side of span n+1
H_Frn_: horizontal component of the resultant tensile force at intermediate support between span n and span n+1
H_m_: horizontal component of the mainline tension
H_Sn_: horizontal component of the skyline tensile force at span n
L_0_: entire unstretched length
L_0n_: unstretched length of span n
L_Gn_: length of guyline
L_Gn0_: unstretched length of guyline
L_Gn1_: slanted distance between the bottom of the tower to the anchor, projected on the plane of skyline
L_Gn2_: lateral distance from the plane of the skyline to the anchor
L_Gn3_: height of tower
L_m_: length of mainline
L_n_: stretched length of span n
L_Rn_: length of intermediate support assembling cable
n_G_: number of guylines
q_0_: weight per metre length of skyline
q_m_: weight per metre length of mainline
Q_n_: weight of carriage and the additional load at span n
Q_nx_: horizontal part of the resulting force of the load
Q_R_: resultant force of weight of carriage, the additional load and additional pulling/slanted force
R_n_: resultant force at intermediate support
S_0_: mounting tension of skyline
S_Gn_: tension of guyline
S_n_: tension at span n
S_m_: tensile stress of mainline
x_smaxu_: horizontal position of maximum sag of unloaded skyline
x_Qn_: virtual horizontal distance of the carriage from the left side of span n (unsagged carriage on the chord)
x_Qnl_: longitudinal deflected horizontal distance of the carriage from the left side of span n
y_n_: sag of skyline of span n
α_n_: slope of the chord of span n
β_n_: angle of the slope at intermediate support
δ: elastic prolongation
δ_n_: inclination-dependent elastic prolongation at span n
γ_Gn1_: slope of the slanted distance between the bottom of the tower to the anchor, projected on the plane of the skyline
ε: coefficient of thermal expansion
μ: coefficient of friction at intermediate support
ΔT: difference in temperature (K)
Θ: temperature

## References

[1] Bont LG, Church RL. Location set-covering inspired models for designing harvesting and cable road layouts. Eur J For Res. 2018; 137(6): 771–792. doi: 10.1007/s10342-018-1139-7.

[2] Arbeitsgruppe Forst (Germany, Austria, Switzerland). Gefährdungen bei forstlichen Tätigkeiten [Hazards of forestry tasks] [Internet]. 2002 [cited 18 May 2021]. Available from: http://www.kwf-online.org/fileadmin/dokumente/Mensch_Arbeit/gefaehrdungsanalyse.pdf.

[3] AUVA. Unfallstatistik Österreich 2018 [Accident statistics in Austria 2018] [Internet]. 2018 [cited 18 May 2021]. Available from: https://www.auva.at/cdscontent/load?contentid=10008.633448&version=1526981135.

[4] BFW. Leitfaden für gefährliche forstliche Arbeiten bei der WLV [manual for dangerous forestry work at the WLV] [Internet]. 2005 [cited 18 May 2021]. Available from: https://bfw.ac.at/rz/document_api.download?content=bfw_leitfaden_wlv_2005_2.pdf.

[5] Kuratorium für Waldarbeit und Forsttechnik e.V. Unfallstatistik–Zeitreihen der Länder [Accident statistics] [Internet]. 2021 [cited 18 May 2021]. Available from: https://www.kwf-online.de/index.php/wissenstransfer/unfallstatistik/352-zeitreihen-der-laender.

[6] SUVA. Unfallstatistik Schweiz [Accident statistics in Switzerland] [Internet]. 2021 [cited 18 May 2021]. Available from: https://unfallstatistik.ch/d/neuza/Suva_Kl_d/WirtKl_BUV_42B.pdf.

[7] Tsioras PA, Rottensteiner C, Stampfer K. Analysis of Accidents During Cable Yarding Operations in Austria 1998–2008. CROJFE. 2011; 32(2): 549–560.

[8] Spinelli R, Magagnotti N, Cosola G, et al. Skyline tension and dynamic loading for cable yarding comparing conventional single-hitch versus horizontal double-hitch suspension carriages. IJFE 2021; 0:1–11. 10.1080/14942119.2021.1909322.

[9] Bont LG, Heinimann HR. Optimum geometric layout of a single cable road. Eur J For Res. 2012; 131:1439–1448.

[10] Mologni O, Marchi L, Lyons CK, Grigolato S, Cavalli R, Röser D. Skyline Tensile Forces in Cable Logging: Field Observations vs. Software Calculations. CROJFE. 2021; 42(2):227–243.

[11] Barat I, Plawinski W. Kabelkrane [Cable cranes]. Berlin: VEB-Technik-Verlag; 1956. p. 63ff.

[12] Zweifel O. Seilbahnberechnung bei beidseitig verankerten Tragseilen [Cable way calculation for suspension ropes anchored on both sides]. Schweizerische Bauzeitung. 1960; 78(1):1–4 and (2):15–20.

[13] Pestal E. Seilbahnen und Seilkrane für den Holz- und Materialtransport [Cable crane and cable yarders for wood and material transport]. Vienna: Verlag Georg Fromme & Co; 1960. p. 262.

[14] Irvine HM. Cable Structures. New York: Dover Publications Inc.; 1981. p. 16–22.

[15] Czitary E. Seilschwebebahnen [Funiculars]. Vienna: Springer Verlag; 1961, doi: 10.1007/978-3-7091-8081-5

[16] Brown C, Sessions J. The standing skyline: A maximum log load solution procedure. For Sci. 1996; 42(2):220–227.

[17] Spinelli R, Marchi E, Visser R et al. Skyline tension, shock loading, payload and performance for a European cable yarder using two different carriage types. Eur J For Res. 2017; 136: 161–170. 10.1007/s10342-016-1016-1.

[18] Such M, Jimenez-Octavio JR, Carnicero A, Lopez-Garcia O. An approach based on the catenary equation to deal with static analysis of three dimensional cable structures. Eng. Struct. 2009; 31:2162–2170. 10.1016/j.engstruct.2009.03.018.

[19] Chunjiang W, Renpeng W, Shilin D, Ruojun Q. A new catenary cable element. Int J Space Struct. 2003; 18:269–275.

[20] Hauska L. Das forstliche Bauingenieurwesen [Forestry civil engineering]. Volume 1: Riesanlagen und Seilbahnen (cableways). Vienna, Leipzig: Verlag Carl Gerolds Sohn; 1933.

[21] Stephan [forename unknown]. Die Drahtseilbahnen [cable ways]. Polytechnisches Journal. 1904; 319:706–709.

[22] Findeis R. Rechnerische Grundlagen des Baues von Drahtseilbahnen [mathematical basics to build cable cranes]. Vienna: Verlag Deuticke; 1923. p. 19.

[23] ess’waer Informatik. seilkranPROjekt [Cableway-computation-software] [Internet]. 2019 [cited 18 May 2021]. Available from: http://www.ewi.cc/seilkranprojekt.html.

[24] Feyrer K. Drahtseile [steel cables]. Berlin, Heidelberg, New York: Springer Verlag; 1994, p. 256ff.

[25] Stüssi F. Zur Theorie des Tragseils bei Militärseilbahnen [Theory of military cable cranes]. Technische Mitteilungen für Sappeure, Pontoniere und Mineure. 1937.

[26] Carson W, Mann C. A technique for the solution of the skyline catenary equations. USDA Forest Service Research Paper. 1970; PNW-110.

[27] Carson W, Mann C. An analysis of running skyline load path. ASDA Forest Research Paper. 1971; PNW-120.

[28] Carson W. Analysis of the single cable segment. For Sci. 1977; 23:238–252.

[29] Falk GD. Predicting the payload capability of cable logging systems including the effect of partial suspension. USDA Forest Service Research. 1981; Paper NE 479.

[30] Kendrick D, Sessions J. A solution procedure for calculating the standing skyline load path for partial and full suspension. For Prod J. 1991; 41(9):57–60.

[31] USDA—US Forest Service Pacific Northwest Forest Products Programs and Software webpage. Skyline XL—a Microsoft Excel Workbook software tool (Version 18) [Internet]. 2019 [cited 18 May 2021]. Available from: https://www.fs.usda.gov/detail/r6/landmanagement/resourcemanagement/?cid=fsbdev2_027048.

[32] Irvine HM. Energy relations for a suspended cable. Q J MECH APPL MATH. 1980; 33(2):227–234.

[33] Dupire S, Bourrier F, Berger FE. Predicting load path and tensile forces during cable yarding operations on steep terrain. J For Res. 2015; 21:1–14. doi: 10.1007/s10310-015-0503-4

[34] Dupire S, Bourrier F, Berger FE. CableHelp: A numerical tool to optimize the set-up of a standing skyline and improve cable yarding planning. IUFRO World Congress. Salt Lake City. 2014.

[35] Python Software Foundation. Python Language Reference [Internet]. 2021 [cited 18 May 2021]. Available from: http://www.python.org.

[36] Moré JJ, Garbow BS, Hillstrom KE. User guide for MINPACK-1. 1980. CM-P00068642.

[37] Czichos H, Henneke M. Hütte–Das Ingenieurwissen [knowledge of engineering]. Berlin: Springer Vieweg; 2012. Volume 34, p. A40. doi: 10.1007/978-3-642-22850-6

[38] Knobloch C. Entwicklung und kombinierte Verwendung eines Portalharvesters und eines mobilen Seilkransystems in forstliche Verfahren zur vollmechanisierten Holzernte auf befahrungssensiblen Standorten [Development and combined use of a portal harvester and a cable yarder system for flat stands in forestry processes for fully mechanized timber harvesting in sensitive, flat forest stands]. Doctoral dissertation, Technische Universität Dresden. 2017.

